# Supplementary material for: Identification of photoperiod-induced specific miRNAs in the adrenal glands of Sunite sheep (Ovis aries)
Source: Front Vet Sci. 2022 Jul 22;9:888207. doi: 10.3389/fvets.2022.888207 (PMC9354845; doi:10.3389/fvets.2022.888207)
Supplement: Supplementary file 1 [file Data_Sheet_1.ZIP › Supplementary file/miRDeep2 file.zip!/miRDeep2 file/Know_Structure/SPLP42A3/oar-mir-23a.pdf]

miRBase precursor : oar-mir-23a
Total read count : 22485
oar-miR-23a read count : 11257
oar-miR-23b read count : 11226
remaining reads : 2

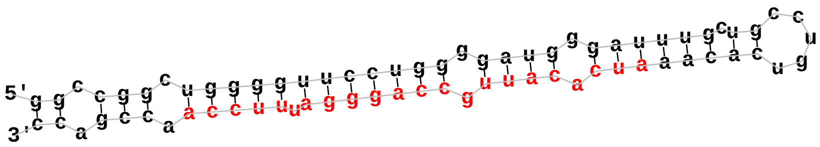

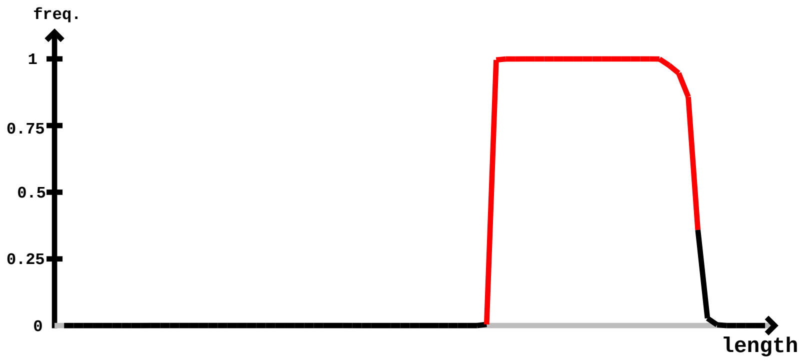

oar-miR-23a
oar-miR-23b

```
5'- ggccggcuggggguuccuggggaugggauuugcugccugucacaaaucacauugccagggauuuccaaccgacc   -3'    exp
    ((.(((.((((((((((((.((((.((((((.((.....)))))))))).)))).))))))).))))).))).))   reads       mm        sample
    .........gggguuccuggggaugggauu............................................   1           0         seq
    .........gggguuccuggggaugggauuu...........................................   1           0         seq
    ............................................aaucacauugccagggau............   2           0         seq
    ............................................aaucacauugccagggauu...........   1           0         seq
    ............................................aaucacauugccagggauuuc.........   6           0         seq
    ............................................aaucacauugccagggauuucc........   31          0         seq
    ............................................aaucacauugccagggauuucca.......   8           0         seq
    .............................................aucacauugccagggau............   3           0         seq
    .............................................aucacauugccagggaC............   1           1         seq
    .............................................aucacauugccagggauu...........   263         0         seq
    .............................................aucacauugcUagggauu...........   1           1         seq
    .............................................aucacauugccagggauuu..........   327         0         seq
    .............................................aucacauugccagggauuuc.........   993         0         seq
    .............................................aucacauugccagggauuucc........   5563        0         seq
    .............................................aucacauugccagggauuucca.......   3711        0         seq
    .............................................aucacauugccagggauuuccaa......   277         0         seq
    .............................................aucacauugccagggauuuccaac.....   28          0         seq
    ..............................................ucacauugccagggauuu..........   1           0         seq
    ..............................................ucacauugccagggauuuc.........   4           0         seq
    ..............................................ucacauugccagggauuucc........   22          0         seq
    ..............................................ucacauugccagggauuucca.......   9           0         seq
    ..............................................ucacauugccagggauuuccaac.....   3           0         seq
    ................................................acauugccagggauuucc........   2           0         seq
    ................................................acauugccagggauuucca.......   1           0         seq
```
